# Electromyography Parameters to Discriminate Hand Osteoarthritis and Infer Their Functional Impact

**DOI:** 10.3390/s24206706

**Published:** 2024-10-18

**Authors:** Verónica Gracia-Ibáñez, Mahdi Mohseni, Angela E. Kedgley, Néstor J. Jarque-Bou, Pablo Granell, Margarita Vergara, Joaquín L. Sancho-Bru

**Affiliations:** 1 Department of Mechanical Engineering and Construction, Universitat Jaume I, 12071 Castelló de la Plana, Spain; 2 Department of Bioengineering, Imperial College London, London SW7 2AZ, UKa.kedgley@imperial.ac.uk (A.E.K.); 3 Consorci Hospitalari Provincial de Castelló, Av. del Dr. Clarà, 19, 12002 Castelló de la Plana, Spain

**Keywords:** hand function, hand osteoarthritis, electromyography, diagnosis, discriminant analysis, diagnosis

## Abstract

Surface-electromyography (sEMG) allows investigators to detect differences in muscle activation due to hand pathologies. However, its use as a functional indicator and the challenges related to the required normalization have not been fully addressed. This study aimed to use forearm muscle sEMG signals to distinguish between healthy individuals and patients with hand osteoarthritis (HOA). sEMG data were collected from seven sensors on the forearms of twenty-one healthy women and twenty women with HOA during the Sollerman test. Amplitude-based parameters (median and range) were normalized using three methods: maximum signals during Sollerman tasks (MAX), during maximum voluntary contraction tasks (MVC), and during maximum effort grasping (GRASP). Waveform parameters (new-zero-crossing and enhanced-wavelength) were also considered. MVC and GRASP resulted in higher values in patients. Discriminant analysis showed the worst success rates in predicting HOA for amplitude-based parameters, requiring extra tasks for normalization (MVC or GRASP), while when using both amplitude (MAX) and waveform parameters and only Sollerman tasks, the success rate reached 90.2% Results show the importance of normalization methods, highlight the potential of waveform parameters as reliable pathology indicators, and suggest sEMG as a diagnostic tool. Additionally, the comparison of sEMG parameters allows the functional impact of suffering from HOA to be inferred.

## 1. Introduction

Forearm muscle activity recorded with surface electromyography (sEMG) during the performance of different daily or sports activities [1,2,3] or during grasping [4] is increasingly used to analyze muscle activation patterns and to detect alterations due to different pathologies. Studying sEMG parameters of the forearm muscles under pathological conditions may provide insight into how muscles activations perform differently as a result [5,6], providing a better understanding of the effects of pathologies on forearm muscles’ function. Additionally, sEMG parameters might serve as indicators able to identify a pathological condition [4]. The World Health Organization emphasizes the importance of objective functional assessments, which should be based on the ability to perform activities representative of daily living [7]. Therefore, if sEMG parameters are recorded during a sufficient variety of tasks that reflect daily living requirements, any alteration in these parameters might provide a more objective perspective on the impact on kinematics and kinetics of task performance, both from a global functionality perspective and on specific daily tasks.

Osteoarthritis is one of the most common pathologies of the hand. It affects 67% of women aged 55 and older [8]. The base of the thumb is frequently affected, with 35.8% of cases involving just the thumb base. This percentage rises to 65% when multiple joints, including the thumb base, are affected simultaneously [8]. Due to changes in the functionality of the joint surfaces and surrounding structures, and associated pain, changes in the activations of the muscles surrounding the joint are not unexpected [6]. sEMG signals of extensor digitorum communis and flexor carpi radialis muscles during four daily tasks have been used to detect differences between women with hand osteoarthritis (HOA) and healthy individuals [5]. The amplitude of the signals was the key feature, and the signals were normalized to those obtained during maximal voluntary contraction (MVC) tasks. Similarly, sEMG has been measured in both wrist extensor muscles—extensor carpi radialis longus and brevis—and in wrist flexor muscles—flexor carpi ulnaris and flexor digitorum superficialis—during four daily tasks [6]. In this case, signals were normalized to the peak of muscle activation during each task. Both studies [5,6] identified differences attributable to HOA in a few specific tasks. However, both studies have limitations in the number of measured muscles and the number of tasks recorded, in addition to using different normalization methods. 

The use of sEMG signal amplitude requires normalization when comparing signals between individuals or sessions [9,10]. Key challenges include normalizing sEMG signals and understanding the impact of different normalization methods, especially for patients and pain consideration. The most common normalization method is relative to MVC [3,5], but there is no consensus on tasks to obtain MVCs of all forearm muscles [4,11], as they depend on the muscles of interest [4,11]. A previous study [5] measured only two muscles and used two tasks to elicit MVC, assuming these tasks would reach MVCs. However, not all individuals achieve MVC for a muscle through the same task [11]. Moreover, while collecting MVCs is feasible in healthy populations, it may be too painful or difficult for patients to perform these tasks, resulting in lower recorded muscle activations. Normalizing with submaximal sEMG signals may falsely suggest that patients require higher activation to perform a task [12].

Recommendations for sEMG recording have been developed for the non-invasive assessment of muscles (SENIAM) project and its extensions [12,13,14]. These include advice about sensors, sensor placement procedures, and signal processing. Besomi et al. [12] provided specific recommendations on normalizing amplitude through a Delphi process that evaluated six sEMG normalization methods, though not specifically for the forearm. They suggested collecting MVCs during activities corresponding to tasks of interest, acknowledging that standardized isometric MVCs may be needed in certain contexts. The study did not specify tasks for obtaining MVCs but stressed that validity depends on participants performing at their maximum capacity, advising against its use in patients with pain. Nevertheless, previous works have used normalization using MVC in patients with HOA [4,5].

Alternatively, signal amplitude can be normalized to the maximum (MAX) signal obtained during all recorded tasks for the same participant [1]. However, this does not accurately reflect the level of activation in each task, as maximum effort may not be reached. Additionally, not all individuals use the same proportion of their maximum activation for a task, making comparisons between individuals or sessions invalid unless identical tasks are performed. Besomi et al. [12] suggest that the MAX method is appropriate only for specific interpretations of EMG data, such as patterns, but highlight its frequent misuse, particularly in comparing amplitudes across groups—a misuse that has also been observed in studies with HOA patients [6]. Despite recommendations, both normalization methods (MVC and MAX) continue to be used [5,6,10], likely due to a lack of better alternatives for normalizing sEMG amplitude. To the best of the authors’ knowledge, no prior studies have compared these methods to assess their differences in detecting abnormalities in patients with HOA.

Some works suggest the possibility of using other forearm sEMG parameters that do not rely on signal amplitude but on the signal waveform [15]. In a previous work [4], sEMG signals from the forearm were recorded while participants performed their maximal force in different grasp types. Waveform parameters were analyzed to determine if they could be used as indicators for pathologies such as HOA: new zero crossing (NZC), enhanced wavelength (EWL), and enhanced mean absolute value were trialed due to their efficiency and simplicity [16,17,18]. These parameters were scaled to 0–1 using the MAX values and analyzed along with the muscle activity (amplitude) normalized using the MVC as potential indicators of HOA. The results demonstrated that the sEMG parameters of forearm muscles were affected by HOA and could successfully discriminate the pathology, raising the question of whether parameters based on daily activities would be similarly impacted by HOA and capable of discriminating the pathology. Although waveform parameters are intended for comparing participant groups, rescaling to a 0–1 range, which normalizes values to the MAX value, was considered beneficial by Besomi et al. [12]. This scaling method was found to be particularly useful for examining activation pattern parameters, as it helped reduce inter-subject and inter-muscle differences in amplitude. While rescaling NZC may just simplify the interpretation of results, it is essential for parameters like EWL, as it effectively minimizes inter-subject differences in amplitude.

It would be advantageous for patients and researchers if sEMG parameters could be utilized as functional indicators of HOA pathology during the performance of representative daily life tasks. This proposal is explored in this paper by comparing sEMG parameters during the entire Sollerman hand function test (SHFT) between groups of healthy women and women with HOA. The SHFT consists of twenty daily tasks representing daily living activities [19]. Combining these data with the performance of each specific SHFT task and comparing between the groups could help assess the impact of HOA on hand functionality. In the absence of a better method and given that amplitude-based sEMG parameters require normalization, this study proposes using both aforementioned normalization methods (i.e., MAX and MVC) in HOA patients to evaluate the real effects and differences associated with each approach. For MVC, two different approaches are used to obtain activations during MVC The first is the common method of performing isometric tasks targeting each muscle [4,11]. The second is a novel method of performing maximal force during grasp types that are representative of functionality [20]. Hereafter, we will refer to these three methods, as well as the values used for signal normalization, as MAX, MVC, and GRASP. In addition to amplitude-based parameters, waveform-based parameters are compared with the same objectives. Unlike amplitude-based parameters, waveform-based parameters do not face the same normalization limitations. sEMG waveform-based parameters might also be used as functional indicators of HOA and provide additional insights into the underlying causes of different behaviors observed in HOA patients at the functional level. Based on previous results [4], we hypothesize that sEMG parameters that differ between groups might also appear during the recording of functional tasks. This might enable the development of discriminant formulas for the early prediction of HOA based on these parameters. This work aims to identify new formulas based on sEMG recorded during the SHFT, which might be considered along with previous results for future deep studies oriented to helping assessment in clinical settings.

In short, this work has three objectives: (i) To examine the discriminative capability of sEMG parameters during the performance of all tasks of SHFT as functional indicators of HOA. To achieve this, we analyzed whether differences existed between recordings from healthy women and women with HOA. These parameters were amplitude-based using three different bases for normalization (MAX, MVC and GRASP) and waveform-based parameters. (ii) To analyze the differences in sEMG parameters between groups per each task to infer the functional impact of suffering HOA. (iii) To propose a series of discriminant formulae for the early prediction of HOA.

## 2. Materials and Methods

### 2.1. Experimental Study

Twenty-one women (47 ± 11 years) with no history of hand pathology or injury (healthy group) and 20 women (71 ± 9 years) with diagnosed HOA (patients’ group) volunteered to take part in this study. All participants signed informed consent, and the experiments had a favorable report from the Hospital Ethics Committee and the Human Research Ethics Committee (CEISH; reference number: CD/27/2022). Specific informed consent for online open-access publication was obtained to acquire photos that could enable the identification of participants. Participants with HOA were recruited by clinicians from hospital patients, representing different stages of the disease and levels of compromise, with none having undergone surgery. The experiment was framed within the collaboration agreement signed with the *Consorci Hospitalari Provincial de Castelló* (9242/2021). Healthy participants were recruited from the research team, university staff, their relatives, and students. For participants with HOA, inclusion criteria were the presence of HOA without prior surgery. For healthy participants, inclusion criteria required no history of neuromuscular problems or upper arm injuries. Aiming to reduce the age difference between groups as much as possible, but considering the huge difficulty in ensuring a group of women with a mean age of 70 years who were free of HOA without using imaging diagnostics, the focus was women aged 45–60 with no symptoms of HOA [21].

**Figure 1:**
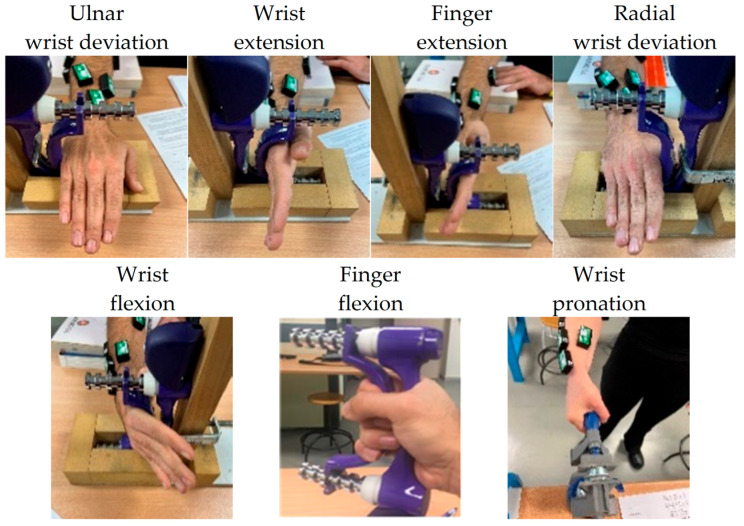
Movements for obtaining maximal voluntary contractions.

**Figure 2:**
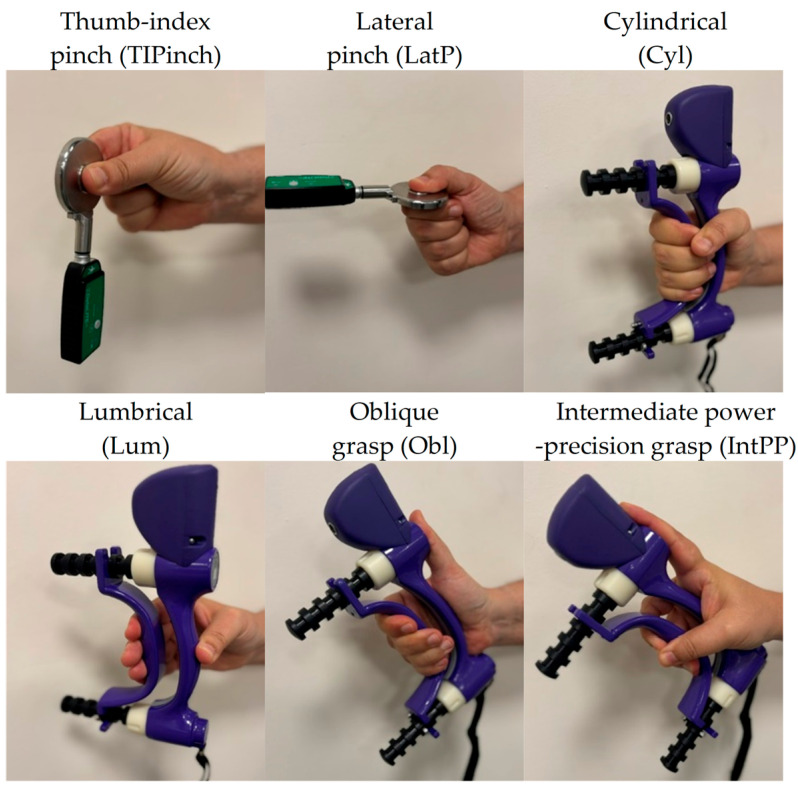
Recording maximal voluntary force in six grasp types relevant for functionality [20].

Muscle activity was recorded using an eight-channel sEMG device from Biometrics Ltd. (Newport, UK), sampling at 1000 Hz. Integral dry reusable sEMG wireless electrodes (LX230) were used for this purpose. The electrodes were secured using double-sided die-cut tapes (T350) on seven locations that captured the representative activation during daily activities according to previously published work [22]. The skin was shaved and cleaned with alcohol. Precise marking for electrode locations was established, following the methodology outlined in [22]; although, the process was facilitated by using an elastic mesh, accurately positioned on the forearm with the guidance of five easily identifiable anatomical landmarks. These landmarks were the ones employed in [22] to draw a grid on the skin, dividing the forearm into zones. The use of the mesh with the grid already drawn on it avoided the need to draw it directly on the skin. These sensor locations capture muscle activation synergies during activities representative of daily functionality and capture signals during the following movements according to previous work [22]: (1) wrist flexion and ulnar deviation (WF&UD), (2) wrist flexion and radial deviation (WF&RD), (3) digit flexion (DF), (4) thumb extension and abduction/adduction (TM), (5) finger extension (FE), (6) wrist extension and ulnar deviation (WE&UD), (7) wrist extension and radial deviation (WE&RD). sEMG signals were recorded while performing three sets of tasks. First, participants performed a series of maximal efforts in wrist flexion/extension, radial/ulnar deviations, and pronation, and fingers’ flexion/extension to elicit MVC (Figure 1). Second, they performed a series of maximal pinch and grip forces to obtain maximal voluntary force while performing relevant grasp types for functionality (Figure 2). Finally, participants performed the defined tasks of SHFT, which are based on the most common hand grips and consist of 20 activities of daily living [19] (Table 1). Each participant performed movements, grasps and these 20 activities under laboratory conditions following the test instructions strictly and using real objects.

### 2.2. Data Analysis

The data analysis was divided into two main parts: amplitude-based parameters and waveform-based parameters. An additional discriminant formula, considering both amplitude and waveform-based parameters that did not require MVC registration, was conducted.

#### 2.2.1. sEMG Amplitude Parameters Normalized Using Three Methods: Healthy vs. HOA Women for Complete SHFT

All signals were filtered using a 4th-order Butterworth band-pass filter with a cut-off frequency between 25 and 500 Hz and a 4th-order Butterworth band-stop filter with a cut-off frequency between 49.5 and 50.5 Hz, using zero-phase filtering. They were then rectified and smoothed by Gaussian smoothing [22]. The sEMG signals of all recordings of each SHFT task for each participant were resampled to 1,000 frames in length.

To normalize the data, three methods were used based on dividing the signal by its maximum values: (i). MAX was calculated as the maximum signal from each sensor for each participant during the entire SHFT; (ii). MVC was calculated as the maximum signal from each sensor considering the maximum force in the seven movements of Figure 1; and (iii). GRASP was calculated as the maximum signal from each sensor considering the maximum force exerted when performing the six grasp types in Figure 2.

First, maximal values from sEMG signals (in mV), once filtered, rectified, and smoothed, were obtained to enable normalization according to the aforementioned three methods. The values were compared. Repeated measures analysis of variance (RMANOVA) was performed to check for differences between methods, with factors: groups (healthy/patients) and sensors. Post-hoc (Tukey’s B) analysis was performed for sensors. RMANOVA and post-hoc analysis were also conducted separately for each group (patients and healthy women).

Then, the effect of group on amplitude-based parameters was computed. The filtered signal, resampled to 1000 frames, was normalized in three ways by dividing it by the maximum values previously obtained using the MAX, MVC, and GRASP methods for each participant, each task, and each sensor. After normalization, two representative parameters of the 1000-frame signal were obtained: the median and range (max–min). These parameters were averaged across tasks to get two representative metrics for the complete SHFT per sensor and per participant for each normalization. These two parameters per sensor were the ones used in statistical analysis: analysis of variance for variables with normal distribution, according to Shapiro–Wilks test, and Kruskal–Wallis for variables with non-normal distributions. The dependent variable was the parameter (median and range) in each sensor, and the factor was the group (healthy/patient).

#### 2.2.2. sEMG Amplitude Parameters Normalized Using Three Methods: Healthy vs. HOA Women for Each SHFT Component

The computational approach described in Section 2.2.1 to compare sEMG amplitude parameters between groups was applied to each task of the SHFT to determine whether the functional impact of suffering from HOA can be inferred by identifying the tasks where patients show differences.

#### 2.2.3. Discriminant Formulae Based on Amplitude Parameters to Detect HOA

The amplitude parameters in the sensors that were found to differ significantly between participant groups in Section 2.2.1. were used as independent variables in a linear discriminant analysis to obtain a formula able to classify women as healthy or patients with HOA [23]. Condition (patients or healthy) was the categorical variable. Linear discriminant analysis is a robust method regardless of normality assumption; however, variance equality is required [24]. Thus, a Box’s M test was conducted to check data suitability [25]. The stepwise method and Wilks’ lambda (Λ) statistic test [23] were used, with predictor variables entered sequentially. At each step, the Λ statistic was computed, and the variable with the least significant level (below 0.05) was included in the formula. The Λ statistic was then recalculated for the discriminant formula with the predictors added up to that point. A predictor was removed if its significance level exceeded 0.1. Classification ability was assessed using leave-one-out cross-validation [25], determining the method’s sensitivity (percentage of true positive detections) and specificity (percentage of true negative detections).

#### 2.2.4. sEMG Waveform Parameters: Healthy vs. HOA Women for Complete SHFT

sEMG signals of each SHFT task for each participant were filtered using 4th-order Butterworth band-pass filter with a cut-off frequency between 25 and 500 Hz, using zero-phase filtering. For each participant, each task, and each sensor, two parameters that were found to be non-highly correlated in previous work [26] were obtained: *NZC* and *EWL* corresponding to Equations (1) and (2), respectively, where *i* is the frame number, *L* is the signal length, and $x_{i}$ is the sEMG signal (mV) at frame *i*.

(1) $$NZC=\sum_{i=1}^{L}{NZC}_{i}\;\;\;\;\;\;\;\;\;\;{NZC}_{i}=\left\{\begin{array}{l} 1,\;\;\;\;if\;x_{i}>0\;and{\;x}_{i+1}<0\\ or\;\;\;x_{i}<0\;and{\;x}_{i+1}>0\;\;\;\;\\ 0,\;\;otherwise\;\;\;\;\;\;\;\;\;\;\;\;\;\;\;\;\; \end{array}\right.$$

(2) $$EWL=\sum_{i=2}^{L}\left|{\left(x_{i}-{\;x}_{i-1}\right)}^{p}\right|\;\;\;\;\;\;\;\;\;\;p=\left\{\begin{array}{l} 0.75,\;\;\;if\;i\geq 0.2L\;and\;i\leq 0.8L\\ 0.50,\;\;otherwise\;\;\;\;\;\;\;\;\;\;\;\;\;\;\;\;\;\;\;\; \end{array}\right.$$

These parameters, obtained per participant, sensor, and task were then divided by the number of frames and multiplied by 1,000, equivalent to a mean value per second, and enabling comparison despite differences in signal lengths. Then, they were rescaled between 0 and 1 per sensor for each participant according to Equation (3) (*NZC*) and Equation (4) (*EWL*): *k* for each sensor, *max_k_* and *min_k_* depict the maximum and minimum values for sensor *k* of each parameter (*NZC* in Equation (3) and *EWL* in Equation (4)) obtained throughout all the tasks for the participant.

(3) $${NZC}_{k}^{normalized}=\frac{{NZC}_{k}-{NZCmin}_{k}}{NZC{max}_{k}-{NZCmin}_{k}}$$

(4) $${EWL}_{k}^{normalized}=\frac{{EWL}_{k}-{EWLmin}_{k}}{EWL{max}_{k}-{EWLmin}_{k}}$$

To get two representative parameters for the whole SHFT per sensor and participant, the parameters were averaged across tasks. The normality distribution for each variable was checked using a Shapiro–Wilks test. Significant differences were computed through an analysis of variance for variables with a normal distribution, and through the Kruskal–Wallis test for variables with a non-normal distribution. The dependent variable was the parameter (NZC or EWL) in each sensor, and the factor was the group (healthy/patient).

#### 2.2.5. sEMG Waveform Parameters: Healthy vs. HOA Women for Each SHFT Component

The computational approach described in Section 2.2.4 was performed for each task of the SHFT individually.

#### 2.2.6. Discriminant Formula Based in Waveform Parameters (NZC and EWL) to Detect HOA

The discriminant analysis described in Section 2.2.3 was performed on sEMG waveform-based parameters. The parameters in the sensors that significantly differed between samples, obtained in Section 2.2.4., were used as independent variables in the linear discriminant analysis.

#### 2.2.7. Discriminant Formula Based in sEMG Parameters Without Requiring MVC Registration to Detect HOA

A discriminant formula using all parameters that do not require MVC registration was conducted, i.e., amplitude-based parameters normalized with the MAX method and waveform-based parameters. This was achieved by performing the same discriminant analysis as in Section 2.2.3 and Section 2.2.6.

## 3. Results

### 3.1. sEMG Amplitude Parameters Normalized Using Three Methods: Healthy vs. HOA Women for Complete SHFT

**Figure 3:**
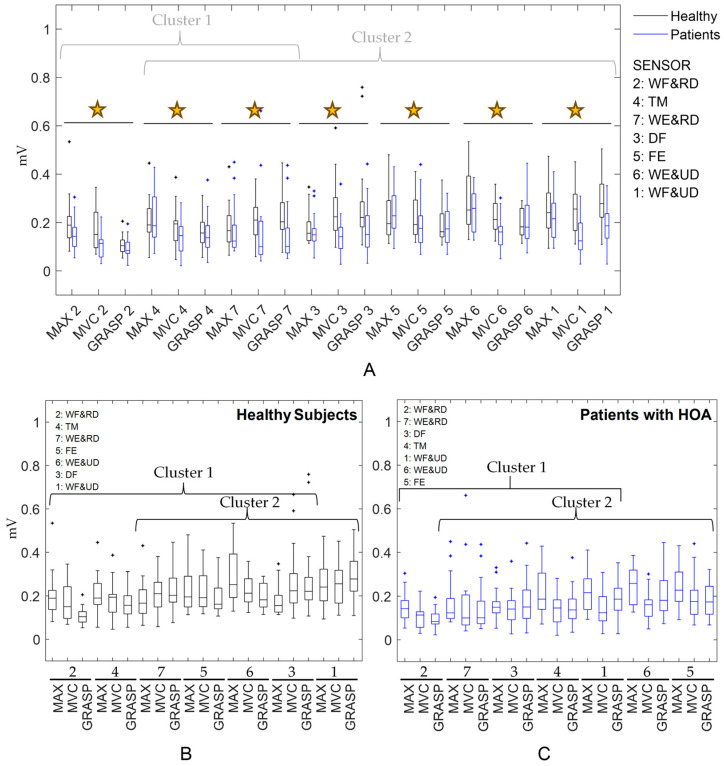
Box and whiskers plots for normalization values of sEMG signals (mV) for healthy (black) and patient (blue) participants according to the maximum (MAX) signal obtained during all recorded tasks for the same participant, those obtained during maximal voluntary contraction (MVC) tasks, and those obtained during maximum effort grasping (GRASP) for each sensor. Results are displayed for (**A**) all participants together, (**B**) healthy participants only, and (**C**) patients with hand osteoarthritis (HOA). Significant differences (*p* < 0.05) between normalizing values are marked with a star (**A**). Clusters in healthy and patient groups are shown with braces. Sensors 1 to 7 are arranged from left to right in ascending order based on sensors with higher mean values showing the clustering obtained from post-hoc analysis. Sensor locations approximately corresponded to 1: wrist flexion and ulnar deviation, 2: wrist flexion and radial deviation, 3: digit flexion, 4: thumb extension and abduction/adduction, 5: finger extension, 6: wrist extension and ulnar deviation, 7: wrist extension and radial deviation [22].

MVC values were not always greater than MAX values (Figure 3A). RMANOVA showed that normalization values depended on the method used, affecting both the participants group and the sEMG sensor. Post-hoc (Tukey’s B), applied to the sensor factor, showed two clusters of sensors with similar mean values when analyzing all participants together (Figure 3A). We hypothesize that the pattern of sensor activation may differ between patients and healthy participants. RMANOVA, conducted separately for each group (patients and healthy women), also revealed differences depending on the normalization method. The post-hoc analysis (Tukey’s B) again identified two clusters of sensors with similar mean values for each group (Figure 3B and Figure 3C, respectively), but the composition of these clusters differed. Notably, while wrist flexion and radial deviation consistently showed the lowest values within cluster 1, wrist flexion and ulnar deviation showed different median values and cluster association: in healthy women, they displayed the highest values, belonging to cluster 2, whereas in patients, they showed lower mean values, belonging to cluster 1.

**Figure 4:**
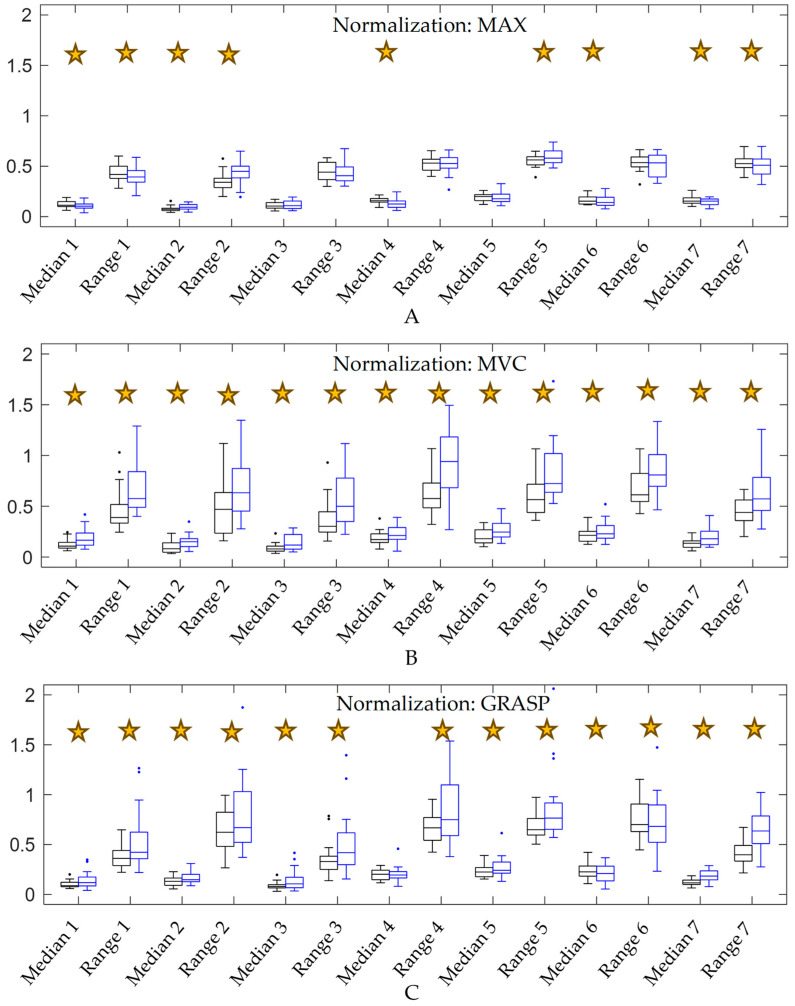
Surface electromyography amplitude parameters: median and range, normalized with three methods according to values used to normalize—(**A**) the maximum (MAX) signal obtained during all recorded tasks for the same participant, (**B**) those obtained during maximal voluntary contraction (MVC) tasks, and (**C**) during maximum effort grasping (GRASP) for each sensor—during Sollerman hand function test tasks (i refers to sensors from 1 to 7) for healthy (black) and patient (blue) groups. Significant differences (*p* < 0.05) are marked with a star. Sensor locations approximately corresponded to 1: wrist flexion and ulnar deviation, 2: wrist flexion and radial deviation, 3: digit flexion, 4: thumb extension and abduction/adduction, 5: finger extension, 6: wrist extension and ulnar deviation, 7: wrist extension and radial deviation [22].

When using MAX normalization, patients showed lower mean values in sensors 1, 4, 6, and 7, and lower range values in sensors 1 and 7, while higher mean values were shown in sensor 2 and higher range values were shown in sensors 2 and 5 (Figure 4A). Patients showed higher median activation and ranges in all sensors using MVC normalization (Figure 4B) and in all sensors except for the mean value of sensor 4 using GRASP normalization (Figure 4C).

### 3.2. sEMG Amplitude ParametersNormalized Using Three Methods: Healthy vs. HOA Women for Each SHFT Component

Patients showed higher median and range values in most tasks and sensors when using MVC to normalize (Table 2). When using GRASP, differences were also observed, also with higher values in patients, but in fewer tasks and sensors than when using MVC. Using MAX normalization, higher values were also found for patients, especially in sensor 2 (wrist flexion and radial deviation), and in some tasks by sensors representing wrist and finger extension (5, 6, and 7, Table 2). Lower median values were found for sensors 1 and 4 (wrist flexion and ulnar deviation and thumb movement), particularly in tasks where these movements were used, such as turning a screw with a screwdriver (task 6), opening the lid of jars (task 10), and in some tasks involving fine manipulation, such as picking up coins (task 1) or opening/closing a zip (task 2).

### 3.3. Discriminant Formulae Based on Amplitude Parameters to Detect HOA

The result of the Box’s M test (>0.05) confirmed equality of the covariance matrix. The discriminant functions obtained (Equations (5)–(7), with variables R for range and M for median, with subscripts MAXi, MVCi, or GRASPi corresponding to the normalization method, and i referring to sensors 1 through 7) were able to predict the assignment of the individuals participating in the experiment with a success ratio after the cross-validation of 70.7% for MAX normalization (70.0% for healthy participants, i.e., specificity, and 71.4% for patients, i.e., sensitivity, Equation (5)), 80.5% for MVC normalization (70.0% for healthy participants, i.e., specificity, and 90.5% for patients, i.e., sensitivity, Equation (6)), and 87.8% for GRASP normalization (85.0% for healthy participants, i.e., specificity, and 90.5% for patients, i.e., sensitivity, Equation (7)). A negative F value means that the formula predicts that the individual has HOA, while a positive value denotes that the individual is healthy.

(5) $$F_{MAX}=8.901\cdot R_{MAX2}-20.053\cdot M_{MAX4}-0.586$$

(6) $$F_{MVC}=-11.308\cdot M_{MVC4}+4.729\cdot R_{MVC4}+2.971\cdot R_{MVC7}-2.931$$

(7) $$F_{GRASP}=7.755\cdot M_{GRASP3}+3.302\cdot R_{GRASP5}-3.184\cdot R_{GRASP6}+4.160\cdot R_{GRASP7}-3.27$$

### 3.4. sEMG Waveform Parameters: Healthy vs. HOA Women for Complete SHFT

**Figure 5:**
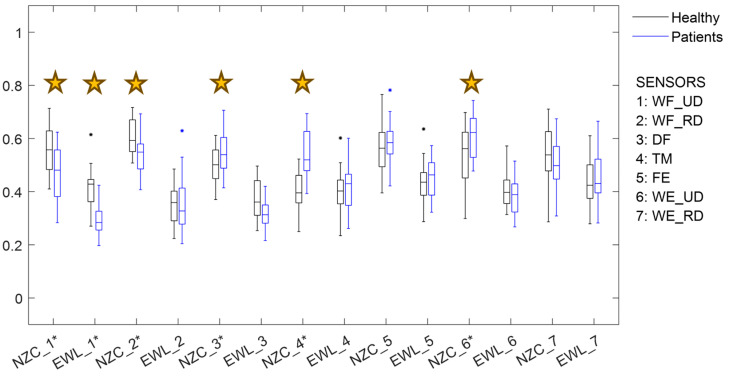
Box and whisker plot of waveform-based surface electromyography parameters: non-zero crossing (NZC) and enhanced wavelength (EWL) for sensors 1 through 7 for healthy (black) and patient (blue) participants during the Sollerman hand function test. Stars indicate a significant difference (*p* < 0.05). Sensor locations approximately corresponded to 1: wrist flexion and ulnar deviation, 2: wrist flexion and radial deviation, 3: digit flexion, 4: thumb extension and abduction/adduction, 5: finger extension, 6: wrist extension and ulnar deviation, 7: wrist extension and radial deviation [22].

Differences were observed between groups, with lower EWL in wrist flexion and ulnar deviation and lower NZC in wrist flexion, with both ulnar and radial deviation. Higher NZC was observed in digit flexion, thumb movement, and wrist extension and ulnar deviation for the patient group when considering the complete set of SHFT tasks (Figure 5).

### 3.5. sEMG Waveform Parameters: Healthy vs. HOA Women for Each SHFT Component

Patients showed significantly lower EWL in wrist flexion and ulnar deviation (sensor 1) in most tasks, as well as in digit flexion (sensor 3) and in wrist extension and ulnar deviation (sensor 6; Table 3). Conversely, patients showed significantly higher EWL in sensors referring to thumb movement (sensor 4), finger extension (sensor 5), and wrist extension and radial deviation (sensor 7) in some tasks, as well as higher NZC in thumb movement (sensor 4) and finger extension (sensor 5). Patients showed lower EWL in sensors referring to wrist flexion–extension and ulnar deviation (sensors 1 and 6) and digit flexion (sensor 4) in many tasks related to fine manipulation, as in tasks: manipulating coins, using a screwdriver, turning nuts onto bolts, or writing with a pen (tasks 1, 3, 6, 7, and 14). Conversely, patients showed higher EWL values in wrist flexion and radial deviation, thumb movement, and finger extension (sensors 2, 4, and 5) in tasks such as cutting Play-Doh with a knife and fork (task 13), folding paper in the air and putting it into an envelope (task 15), and pouring water from a pure-Pak (task 18). In fact, these tasks present higher values in many parameters, especially when cutting Play-Doh with a knife and fork.

### 3.6. Discriminant Formula Based on Parameters NZC and EWL to Detect HOA

The result of the Box’s M test (>0.05) confirmed equality of the covariance matrix. The discriminant function obtained (Equation (8), with variables EWLi and NZCi, where i refers to sensors 1 through 7) was able to predict the assignment of the individuals participating in the experiment with a success ratio after the cross-validation of 87.8%: 85.7% for healthy participants, i.e., specificity, and 90.0% for patients, i.e., sensitivity). Obtaining a negative $F_{Waveform}$ value using the Equation (8) from the ${EWL}_{1}\;and\;{NZC}_{4}$ parameters indicates that the woman is classified as patient, while a positive value indicates she is classified as healthy.

(8) $$F_{Waveform}=-8.738\cdot {EWL}_{1}+9.624\cdot {NZC}_{4}-1.481$$

### 3.7. Discriminant Formula Based on sEMG Parameters Without Requiring MVC Registration to Detect HOA

The discriminant formula obtained (Equation (9)), if all the sEMG parameters that do not need additional recordings to SHFT are included, increases their predictability compared to Equation (8), achieving a success ratio after the cross-validation of 90.2%: 85.7% for healthy participants, i.e., specificity, and 95.0% for patients, i.e., sensitivity).

(9) $$F_{NoMVC}=-4.407\cdot R_{MAX\;2}+14.673\cdot M_{MAX\;4}+\ldots \;\ldots +7.508\cdot {EWL}_{1}-8.740\cdot {NZC}_{4}+1.109$$

## 4. Discussion

The first objective of this study was to evaluate how well sEMG parameters can distinguish between individuals with and without HOA during tasks representative of daily functionality. Differences in both amplitude parameters (median and range) and waveform parameters (NZC and EWL) were found due to the HOA condition. These findings align with previous research, which also observed differences in both amplitude parameters during several daily tasks [5,6] and waveform parameters during maximal force exertion in different grasp types [4]. The novelty of this study lies in obtaining these parameters from a broader range of daily tasks that are representative of the grasps of daily living activities, as well as in the comparison of different normalization methods for amplitude parameters.

The first approach considered amplitude parameters. While MAX and MVC are normalization methods commonly used for amplitude parameters, their use is often discouraged for comparing groups where individuals are affected by pain [12], such as HOA patients. Despite this, both methods are still widely used due to a lack of better alternatives. The use of submaximal efforts, as an alternative, when performing an MVC is challenging, is also discouraged due to the differences in muscle activation patterns, which can lead to invalid comparisons between groups [12]. The comparison of normalization methods will determine the implications of using each method and enable comparisons with previous works. In this work, three normalization methods were compared: MAX, MVC, and GRASP. The latter two are similar, as both require performing additional tasks to elicit MVC from the muscles for normalization. MVC, the commonly used method, involves performing isometric tasks targeting each muscle [4,11], whereas GRASP, a novel method, requires exerting maximal force during grasp types that are representative of functional tasks [20], making it potentially more relevant for this study. Additionally, performing maximal force in various grasp types is easier for patients to understand compared to isometric tasks.

The results showed that MVC and GRASP methods generally produced similar outcomes, both for the complete SHFT (Figure 4) and for each individual task (Table 2). The difficulty of isometric tasks implies that MVC may not be reached, leading to lower values for normalization, which in turn results in greater differences in sEMG parameters using MVC to normalize, compared to GRASP (Table 2).

**Figure 6:**
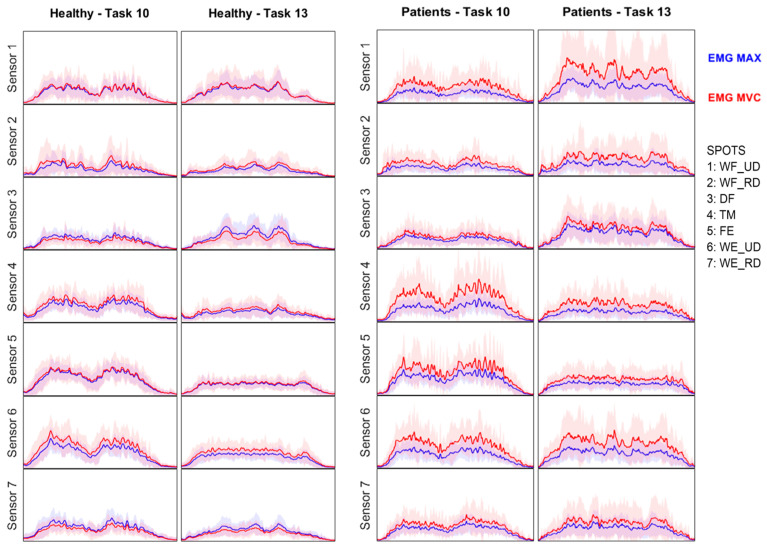
Time evolution of normalized surface electromyography signals (mean across participants with standard deviation shaded), normalized with MAX (blue) and MVC (red) methods for both groups in two specific tasks usually reported as tricky for patients with HOA: unscrewing the lid of jars (task 10) and cutting Play-Doh with a knife and fork (task 13).

MAX and MVC methods yielded significantly different outcomes, highlighting the impact of the chosen method. Differences depended on the normalization method, group, and sensor, as some activities were typically challenging for women with HOA. Two specific tasks usually reported as tricky for patients with HOA were unscrewing the lid of jars (task 10) and cutting Play-Doh with a knife and fork (task 13). Figure 6 shows how, for these tasks, while sEMG signals in healthy women generally appeared very similar when comparing MAX and MVC normalization methods, in women with HOA the signals were much higher using MVC, especially in the thumb (sensor 4) and finger and wrist extension (sensors 5 and 6) during task 10, and in wrist ulnar deviation (sensors 1 and 6) during task 13. Thus, time evolutions of signals presented different shapes between healthy women and women with HOA; but, while in healthy women signal differences between methods were similar across almost all sensors, in women with HOA differences between methods were highly dependent on the sensor.

These differences between MAX and MVC methods likely explain the conflicting findings of Tossini et al. [6] and Bronson et al. [5]. Tossini et al. [6], who used MAX normalization, observed reduced muscle activity in wrist muscles (extensor carpi radialis, flexor carpi ulnaris, and in flexor digitorum superficialis) in individuals with HOA during tasks like writing, cutting with scissors, and opening and closing a bottle. Conversely, Bronson et al. [5] inferred that women with HOA tended to use higher muscle activation both in wrist extensors and flexors (extensor digitorum communis and flexor carpi radialis) during similar but used MVC normalization. The studies monitored different muscles that actuate wrist flexion–extension and used different normalization methods, which suggests that the conflicting results may be due to wrist radial–ulnar deviation or to the normalization method. The present paper (Table 2) showed lower activation in wrist flexion and ulnar deviation (sensor 1) during writing (task 14) when using MAX normalization, which agrees with the findings of Tossini et al. [6]. When using MVC normalization (Table 2), wrist flexion and radial deviation (sensor 2) showed significantly higher activation in patients with HOA across many tasks, including using a key (task 8), along with higher extension parameters (sensors 5, 6 and 7) as seen during writing (task 14). These results align with those of Bronson et al. [5]. Hence, these differences are likely attributable to the normalization method. When all tasks were considered, the results were consistent: using MAX normalization (Figure 4A), patients showed lower parameters in wrist flexion and ulnar deviation (sensor 1) but higher parameters with radial deviation (sensor 2), while extension parameters were lower in both directions. However, using MVC normalization (Figure 4B), generally higher parameters were observed. Using MAX normalization, amplitude differences may not reflect actual differences in muscle activation, as they are not referenced to MVCs. With MVC normalization, results in HOA patients should be interpreted cautiously due to potential pain influence. However, both normalization methods can distinguish differences due to HOA and serve as functional indicators, provided conclusions are drawn carefully.

The RMANOVA applied to normalization parameters (Figure 3) provided valuable insights into the robustness of normalized amplitude parameters. For example, post-hoc analysis showed that wrist flexion and ulnar deviation (sensor 1) clustered differently in healthy women and patients, while wrist flexion and radial deviation (sensor 2) was clustered similarly in both groups, consistently presenting the lower values. Thus, the activation pattern related to sensor 2 is consistent across both groups, unlike sensor 1. According to Besomi et al. [12], the robustness of interpretation may be strengthened by the convergence of findings from two normalization methods. While sensor 2 showed higher activation in patients (Figure 4) using the three methods (MAX, MVC, and GRASP), reinforcing the robustness of this interpretation, sensor 1 shows lower activation in patients using MAX normalization and higher activation using MVC or GRASP normalization.

It would be logical to assume that if MVCs (both in MVC and in GRASP method) are measured best by selecting tasks or actions that are intended to elicit the MVC of the muscles, they would typically yield normalization values higher than MAX, at least in healthy participants. However, Figure 3A shows that MVC and GRASP values for normalizing are not always higher than MAX values. Therefore, the specific actions performed to normalize with MVC should always be reported and taken into consideration when interpreting results.

The second approach, involving the use of waveform-based parameters, also demonstrated potential as a functional indicator capable of discriminating HOA pathology, without the limitations associated with amplitude-based parameters that require normalization. This aligns with previous research [4], where significant differences in waveform parameters were found, even though participants exerted maximal force in six different grasp types rather than during functional tasks. However, during the execution of maximal force in grasp types [4], a higher number of differences in these parameters were found between healthy women and those with HOA than during the performance of functional tasks (Figure 5). These discrepancies may be attributed to the challenges that patients with HOA face in exerting maximal force due to pain, leading to altered activation patterns. In contrast, daily tasks require less force, allowing activation patterns to more closely resemble those of healthy participants.

To ensure the comparability of results with previous studies, sensor placement is as crucial as sEMG parameter selection. Two approaches can be considered in sensor placement: locating the anatomical points that maximize the signal of a specific muscle [5,6] or locating points that produce signals that are representative of function [22]. The presence of crosstalk implies that no matter how strictly guidelines for sensor placement are followed, there will always be an element of approximation in the interpretation of the causes that generate different signals [27]. Only an intramuscular signal would guarantee a pure signal from a specific muscle [28], thus enabling that differences in signal are attributable to a single muscle; however, it also has associated drawbacks, including being invasive. The second approach for sensor placement, which is used in this study, assumes by definition that the sensors are capturing the signal from multiple muscles. Furthermore, the loss of signal due to the movement of surface electrodes relative to the underlying muscles during pro-supination [29] is minimized with this second approach, as the electrode locations were specifically selected to ensure that representative signals of functionality were not lost [22]. Additionally, if the goal is to assess the ability to use the signal to discriminate the presence of HOA, it does not necessarily need to correspond to the signal from a specific muscle.

The second objective of the work was to infer the impact of HOA on functionality through sEMG parameters per task. Patients showed higher values for wrist flexion and radial deviation (sensor 2) in tasks such as cutting with a knife and fork, folding a paper and putting it into an envelope in the air, or during pouring tasks (tasks 13, 15, 18, 19, and 20) across all normalization methods. This consistency suggests that in these cases, patients likely experienced higher activation, which requires greater effort. The higher activation observed implies a greater effort in these tasks, which reflects the impact on functionality experienced by patients with HOA. This may be due to the need for grasp stabilization affecting the thumb base joint when folding paper in the air, combined with the added challenge of maintaining a forced, difficult posture with the wrist flexed and in radial deviation while exerting force to complete the task, as seen when cutting with a knife and fork. Contrarily, patients showed a lower amplitude in wrist extension and ulnar deviation (sensor 6) in tasks: opening/closing a zip, using a screwdriver, and opening a jar lid (tasks 2, 6 and 10), with both MAX and GRASP normalizations. Due to the setup of the SHFT, when performing these three tasks, an extreme wrist extension posture with occasional ulnar deviation (sensor 6) occurs, which could be a source of pain for HOA patients. This may lead patients to exert less force conservatively in order to avoid pain.

Waveform-based parameters (Table 3) can convey additional information. Patients showed significantly lower EWL in wrist flexion and ulnar deviation (sensor 1) in most of the tasks, but also in digit flexion (sensor 3) and in wrist extension and ulnar deviation (sensor 6), suggesting a reduced variability or complexity in muscle activity. This could reflect diminished muscle function, indicating being less capable of dynamically adjusting during tasks, or an increased joint stiffness. This is presented in tasks implying fine manipulation that can be more complicated for patients [30], including opening/closing a zip, using a screwdriver, opening a jar lid, and writing (tasks 2, 6, and 10), which also presented reduced amplitude with MAX normalization in wrist extension and ulnar deviation (sensor 1) and in thumb movement (sensor 4). Higher values in NZC implied worse motor control with more unstable muscle activation, and higher values of EWL implied muscles working harder to stabilize the grasp, such as when cutting with a knife and fork (task 13), which was the most challenging task reported by patients with HOA [30]. Higher EWL values in wrist extension and radial deviation (sensor 2) were found in tasks such as cutting with a knife and fork, folding a paper and putting it into an envelope in the air, or during pouring tasks (tasks 13, 15, 19, and 20), consistent with higher activation found in amplitude-based parameters. Higher NZC values appeared in thumb movement (sensor 4) during turning nuts onto bolts, opening a jar lid, folding paper in the air, and pouring water from a pure-Pak (tasks 7, 10, 15, and 18). Even though only these four tasks showed higher NZC values in sensor 4, these differences must be relevant, since the NCZ parameter for sensor 4 was included in the discrimination formula (Equation (8)).

The third objective, which involved proposing discriminant formulas for the early prediction of HOA, was successfully achieved using both amplitude-based parameters with different normalization methods (Equations (4)–(6)) and waveform-based parameters (Equation (8)). In Equation (4), parameters from the sensor corresponding to wrist flexion and radial deviation, along with the median value in thumb movement, were required. For Equations (5) and (6), more parameters were needed, but the success rates also increased. However, applying these equations in clinical settings may be challenging due to the additional tasks or maximal force exertion required for normalization, as in [4]. In contrast, Equation (8) requires only two parameters, meaning that two sensors recording while an individual completes the SHFT, without the need for additional tasks for normalization, would suffice. By including all parameters based solely on the SHFT recordings, the success ratio increases slightly to 90.2%, requiring only three sensors (numbers 1, 2, and 4, as shown in (Equation (9)). Most importantly, this approach avoids the need for additional tasks to obtain MVCs, which can be challenging for patients. Nevertheless, it is not as easily applicable for clinical use as the discriminant formula found in a previous study, which showed that measuring maximum lumbrical grip force alone was sufficient to detect early HOA with a good success rate [31]. Testing both methods with more extensive populations to check their feasible applicability for clinical assessment, perhaps adding grasp forces to sEMG metrics, should be conducted.

Finally, it should be noted that, in addition to patients with HOA having reduced grasp force across grasp types due to forearm muscle alterations caused by HOA [31], they also exhibit impaired intrinsic muscle forces [21]. However, little is known about the role of intrinsic muscles in daily activities, likely due to the challenges of measuring these muscles with sEMG [32]. While some studies have used intramuscular EMG to analyze activation patterns and grasp stability [33,34], they have not focused on assessing alterations during daily tasks in patients with HOA. Future research should include sEMG recordings from intrinsic muscles during daily tasks to determine if differences attributable to HOA can also be observed.

## 5. Conclusions

In this study, we demonstrated the effectiveness of using both amplitude-based and waveform-based sEMG parameters as reliable indicators of hand osteoarthritis. Our findings revealed differences exist in both types of parameters when comparing patients with HOA with a healthy population, and highlighted the importance of normalization methods. We also provided insights into specific functional impairments associated with HOA, such as increased activation of wrist flexors and radial deviators during tasks like cutting or pouring to maintain grasp stability. Finally, we developed discriminant formulas that could potentially be used for early detection of the disease, given the high success ratios obtained and the varying degrees of severity of HOA within the patient sample. The combined use of both amplitude-based and waveform-based parameters with MAX normalization proved to be the most effective approach, achieving a success rate of 90.2% without the need for additional MVC tasks. These results suggest that sEMG could be a valuable diagnostic tool in the future. Future work should include sEMG recordings of both intrinsic and extrinsic muscles in large populations, and the use of machine learning techniques to improve HOA diagnosis.

## Figures and Tables

**Table 1:** Tasks conforming the Sollerman hand function test (corresponding to daily activities) [19].

**Tasks Conforming the Sollerman Hand Function Test**
Pick coins up from flat surface, put into purses mounted on wall
2. Open/close zip
3. Pick up coins from purses
4. Lift wooden cubes over edge 5 cm in height
5. Lift iron over edge 5 cm in height
6. Turn screw with screwdriver
7. Pick up nuts and turn them until completely screwed onto bolts
8. Put key into Yale lock, turn 90°
9. Turn door-handle 30°
10. Unscrew the lid of jars
11. Do up buttons
12. Put Tubigrip stocking on the other hand
13. Cut Play-Doh with a knife and fork
14. Write with pen
15. Fold paper, put into envelope
16. Put paper-clip on envelope
17. Lift telephone receiver, put to ear
18. Pour water from Pure-Pak
19. Pour water from a jar
20. Pour water from a cup

**Table 2:** Comparison of healthy vs. patients’ surface electromyography amplitude-based parameters (i.e., median and range) during Sollerman hand function test tasks (in columns) for each sensor (in rows) with three normalization methods (MAX, MVC, and GRASP). The blocks with significant differences are highlighted with a sign − (green) or + (red) depending on whether the mean value across patients of each parameter (median and range) was lower (-) or higher (+) than median value across healthy participants.

Sensor	Parameter	Normalization Method	Task Number																			
			01	02	03	04	05	06	07	08	09	10	11	12	13	14	15	16	17	18	19	20
1	Median	MAX		-				-	-			-				-						
	Range			-	-		+					-			+							
2	Median								+		+		+	+	+		+			+	+	+
	Range										+		+	+	+		+	+	+	+	+	
3	Median																			+		
	Range				-																	
4	Median		-	-	-			-	-		-	-							-			
	Range		-		-		-							+								
5	Median				-											+						
	Range				-									+			+					+
6	Median			-				-				-										
	Range			-											+						+	
7	Median				-								-									
	Range				-		-								+							
1	Median	MVC			+	+	+			+			+	+	+		+	+	+		+	+
	Range		+			+		+	+	+	+		+	+	+	+	+	+		+	+	+
2	Median					+		+	+	+	+		+	+	+		+	+		+	+	+
	Range							+			+		+	+	+		+		+	+	+	+
3	Median			+	+	+	+		+	+	+		+	+	+		+	+	+	+	+	+
	Range					+	+		+		+		+	+	+	+	+		+	+	+	+
4	Median														+		+				+	+
	Range					+		+	+	+		+	+	+	+	+	+	+	+	+	+	+
5	Median					+	+		+	+			+	+			+	+		+	+	+
	Range		+			+		+	+	+	+	+	+	+	+	+	+	+	+	+	+	+
6	Median													+	+						+	
	Range					+					+			+	+						+	
7	Median						+		+	+		+	+	+	+		+	+	+	+	+	+
	Range		+			+				+		+	+	+	+	+	+		+	+	+	+
1	Median	GRASP				+	+						+	+	+		+	+				
	Range					+								+	+		+			+		
2	Median													+	+		+			+	+	+
	Range										+				+		+		+	+	+	
3	Median												+	+	+		+	+		+	+	+
	Range					+					+		+	+	+							+
4	Median										+								-			
	Range											+		+			+				+	+
5	Median																			+		+
	Range					+								+		+	+		+	+	+	+
6	Median			-				-				-				-						
	Range			-									-									
7	Median		+	+	+	+	+	+	+	+	+	+	+	+	+	+	+	+	+	+	+	+
	Range		+	+		+		+		+	+	+	+	+	+	+		+	+	+	+	+

**Table 3:** New zero crossing (NZC) and enhanced wavelength (EWL) during Sollerman hand function test tasks (in columns) for each surface electromyography sensor (in rows) for patients and healthy participants. The blocks with significant differences are highlighted with a sign − (green) or + (red) depending on whether the mean value for patients was lower (-) or higher (+) than the median value for healthy participants.

		Sollerman Hand Function Test Task Number																			
		1	2	3	4	5	6	7	8	9	10	11	12	13	14	15	16	17	18	19	20
1	NZC				-					+				+					-		-
	EWL	-	-	-		-	-	-			-			+	-			-	-		
2	NZC					-	-			+			-								
	EWL	-		-							-			+		+		-		+	+
3	NZC											+					+	+	+		
	EWL	-		-			-	-			-			+	-						
4	NZC							+			+					+			+		+
	EWL			-									+	+		+		-	+		+
5	NZC								+	+				+							
	EWL			-												+			+	+	+
6	NZC													+	+			+			
	EWL		-				-	-			-			+	-					+	
7	NZC					-															
	EWL	-		-									+	+		+					

## Data Availability

The data presented in this study are available upon request from the corresponding author.
